# News and Miscellany

**Published:** 1900-04

**Authors:** 


### News and Miscellany.

**Dr. Tullis,** of Quanah, Texas, a member of the live stock Commission, died at Quanah, March 29, ult.

**Dr. J. M. Litten,** an old landmark of Austin, a practitioner of over fifty years in this city, died March 31, ult., aged about eighty.

**Dr. C. F. Norton,** of El Paso, a graduate of Texas Medical College, has been appointed Quarantine Inspector at El Paso, vice Yandall, deceased.

**The Bubonic Plague** is in San Francisco, in Chinatown. The Marine Hospital Service and the California health officials are active in measures to exterminate it.

**The Texas Quarantine** went into effect April 1, (inst.)—a month earlier than usual, on account, it is supposed, of the bubonic plague. By the bye, the existence of the plague in Yucatan is officially denied.

**County Health Officers.**—We have in type a complete list of the County Health Officers in Texas, and have been trying to get a chance to publish it, but have not yet had room for it. It was furnished by Secretary I. J. Jones of the Quarantine department, and will appear soon.

**Dr. W. N. Rogers,** of Waco, Texas, requests us to ask the members of the Texas State Medical Association and visitors who will be present at the meeting of the Association, at Waco, on 24th inst., to visit his sanitarium, "The Provident," and investigate his methods of treatment of drug habits, etc.

**Change of Address of Texas Physicians:**—Dr. C. L. Orr, Kennedy to Waxahachie; Dr. J. B. Ramsay, Emmet to Alto—his old home; Dr. M. B. Grace, Walnut Springs to Seguin; Dr. Percy Baker, Dallas to San Angelo, Texas; Dr. G. L. Robertson, Leander to Victoria, Texas; Dr. Joe Gilbert, Hornsby to Austin, Texas.

**Dr. A. J. G. Devron,** one of the best known and most popular physicians of New Orleans, a scientist, philologist, historian and the best-posted resident of New Orleans in regard to Louisiana in colonial days, died March 26, ult. He had been a sufferer from heart troubles for several weeks, and the organic affliction carried him off in spite of the most enlightened and careful treatment.—*N. O. Picayune.*

---

**The Manufacturers** of an anti-fat preparation are urging ye editor of the "Red-Back" to give their preparation a trial, and offer to send a dollar's worth free, if he will use it. The editor of a leading medical journal up north, who has "Dan'els'" photograph, writes us that we look like we hadn't had a good feed in a year, and says, "Come up and spend a month with me and get a square meal." We "dasn't"; aint used to it.

---

**Dr. Wm. Yandell,** long time quarantine inspector at El Paso, Texas, committed suicide in that city recently. The act is said to be attributed to badly impaired health and despondency. He was well known both to the medical profession and the press, having been for years a leading newspaper editor, and was ex-president of the Texas Press Association. He was a brilliant writer and an accomplished physician. He was the youngest and last living brother of the late Dr. David Yandell, of Louisville, and son of the late Prof. Lunsford Yandell, Sr., of Louisville, Ky.

---

**Christian Science.**—

Dear Christian Science people,
You are a funny folk;
The funniest things you tell us,
And swear they are no joke;

A discovery you've made
Astounding all creation;
Diseases don't exist,
They are just imagination!

Oh what good news you bring,
How much it ought to please us,
That neither ache nor pain
Can any longer tease us!

And yet it's very queer,
And this in you I'm blaming,
That, when there's no disease,
To heal disease you're claiming.

—*The Living Church.*

**Whiskey and Drug Habit.**—Dr. L. V. Weathers, Bracken, Texas, writes the JOURNAL that a few drops of tinct. cinchona dropped far back on the tongue will at once overcome the craving for whiskey in a drinker. He submits also the following formula for both whiskey and morphine craving, and says he has used it successfully:

℞ Ammonium Bromidi..............gr. v

Fl. ext. Belladonnæ

" " Nux Vomica

" " Cannabis Indica aa........ ♏ij

Aquæ.................℥ii, for one dose

To be taken four times a day.

Dr. Weathers gives this for the benefit of the profession and humanity, and scores certain doctors who keep their experiences secret.

